# A Review of HPV-Related Head and Neck Cancer

**DOI:** 10.3390/jcm7090241

**Published:** 2018-08-27

**Authors:** Kazuhiro Kobayashi, Kenji Hisamatsu, Natsuko Suzui, Akira Hara, Hiroyuki Tomita, Tatsuhiko Miyazaki

**Affiliations:** 1 Pathology Division, Gifu University Hospital, Gifu University Graduate School of Medicine, 1-1 Yanagido, Gifu 501-1194, Japan; hern@live.jp (K.K.); y3f3f84d72xsx@yahoo.co.jp (K.H.); nsuzui7@gifu-u.ac.jp (N.S.); ahara@gifu-u.ac.jp (A.H.); 2 Department of Tumor Pathology, Gifu University Graduate School of Medicine, 1-1 Yanagido, Gifu 501-1194, Japan

**Keywords:** human papillomavirus, human cancer, head and neck, reduction therapy

## Abstract

Head and neck squamous cell carcinomas (HNSCCs) arise in the mucosal lining of the upper aerodigestive tract. Tobacco and alcohol use have been reported to be associated with HNSCC. Infection with high-risk human papillomaviruses (HPVs) has recently been implicated in the pathogenesis of HNSCCs. It is now widely accepted that high-risk HPV is a cause of almost all cervical cancers as well as some forms of HNSCCs. HPV-related HNSCCs are increasing. HPV-related HNSCCs and HPV-unrelated HNSCCs differ with respect to the molecular mechanisms underlying their oncogenic processes. HPV-related HNSCCs are known to have a better prognosis response to treatment as compared with HPV-unrelated HNSCCs. Therefore, in recent years, it has been required to accurately discriminate between HPV-related and HPV-unrelated HNSCCs. To diagnose the HPV-related HNSCCs, various methods including *P16* immunohistochemistry, FISH, and genetic analyses of the HPV gene from histopathological and liquid biopsy specimens have been employed. Based on the results of the differential diagnosis, various treatments employing EGFR TKI and low-dose radiation have been employed. Here, we review the involvement of the HPV virus in HNSCCs as well as the molecular mechanism of carcinogenesis, classification, prognosis, diagnostic procedures, and therapy of the disease.

## 1. Introduction

The role of human papillomavirus (HPV) in carcinogenicity was confirmed in 1983 following the cloning of HPV 16 type from cervical carcinoma tissue by Durst and colleagues [1]. It has since become widely accepted that high-risk HPV is a cause of almost all cervical cancers. Many cases of HPV infection are asymptomatic and resolve spontaneously, but cervical cancer may arise in cases of persistent HPV infection of the cervical basal cells [1,2]. As reviewed by Kreimer et al. [3], HPV DNA has been detected by polymerase chain reaction (PCR) in head and neck squamous cell carcinoma arising from various anatomic sites. HPV16 is the predominant HPV type, accounting for 90% of HPV DNA-positive HNSCCs. Various studies involving mainly HPV 16 have shown that viral DNA is diffusely present in tumor cells of whole tumor, and exhibits clonality when detected by in situ hybridization (ISH) [4,5,6]. As shown in several oral and oropharyngeal carcinoma cell lines [7,8,9], the retention of viral DNA during the growth of tumor cells in culture provides further evidence suggestive of viral clonality. 

Various studies have reported that oral and tonsillar epithelial cells can be immortalized by full-length HPV 16 or its E6/E7 oncogenes [10,11,12,13,14]. Additionally, transgenic mouse models have revealed that HPV 16 E6/E7 strongly increases susceptibility to oral and oropharyngeal carcinomas [15]. Although E7 was much more competent in inducing these tumors [15], a clear synergy between E6 and E7 in causing HNSCC was discovered [16]. In an analysis of paraffin-embedded biopsies of 116 cases of laryngeal squamous cell carcinomas by in situ DNA hybridization using 35S-labelled HPV (types 6, 11, 16 and 30) DNA probes, 15/116 (12.9%) tumors were shown to contain the DNA of at least 1 HPV type. HPV 11 was the most frequent DNA type, found in 9/116 (7.8%) of the lesions; HPV 16 was found in 5.2%, and HPV 6, in 4.3% [17]. HPV was recognized as a risk factor for oropharyngeal carcinogenesis by the International Agency for Research on Cancer (IARC) in 2007 [18]. 

A higher frequency of oral sex and a greater number of sex partners are thought to increase the risk of HPV-related cancer in the oropharynx [19,20]. 

The development of a vaccine for the primary prevention of HPV infection subsequently became an urgent worldwide priority. In 2017, Muranaka was awarded the John Maddox Prize for raising public awareness of the efficacy of the HPV vaccine for the prevention of cervical and other cancers (http://senseaboutscience.org/activities/2017-john-maddox-prize/). This review summarizes the involvement of HPV virus, molecular pathological implications, classification and prognosis, and prospects for future treatment in head and neck cancers.

## 2. Involvement of HPV Virus in Head and Neck Cancers

HPV is a DNA virus that infects the skin and mucous membranes. More than 100 types of HPV have been classified to date. Previous studies have examined the role of HPV-related carcinogenesis in uterine cervical cancer. HPV infecting the uterine cervix is divided into high- and low-risk groups. HPV 16, 18, 31, 33, 35, 39, 45, 51, 52, 56, 58, 68, 69, and 73 are classified as high-risk HPV [21], which is estimated to account for almost 100% of cases of cervical cancer, about 90% of cases of anal cancer, and 40% of vulva, vagina, and penile cancer. Additionally, at least 12% of pharyngeal cancer, 3% of oral cancer, and 30–60% of oropharyngeal carcinoma cases are caused by HPV infection [22]. An increase in squamous cell carcinoma of the head and neck has been reported and attracted global attention recently in the world [23]. It is now recognized that there are two types of squamous cell carcinoma of the head and neck. Classically, most oropharynx cancers are hyperdifferentiated and often show keratinization. Squamous cell carcinoma of the head and neck can be of the keratinized or nonkeratinized type. The former occurs most often in elderly males and is associated with smoking and alcohol consumption, but HPV is not involved. Conversely, nonkeratinizing squamous cell carcinoma occurs most commonly at age 40–55 years in men with little exposure to tobacco and alcohol, and HPV DNA is detected as the most characteristic feature [24,25,26,27]. Since the smoking rate in the USA is declining [28], the incidence of HPV-negative tobacco-related oropharyngeal cancer has decreased; however, that of HPV-positive oropharyngeal cancer is increasing [29]. According to repository data from the Surveillance, Epidemiology, and End Results (SEER) program, the prevalence of HPV-negative cancers decreased by 50% from 1988 to 2004, while HPV-positive oropharyngeal carcinoma increased by 225% [30]. In a study by Junor and associates involving patients at the Edinburgh Cancer Center, 41% of head and neck cancers were HPV-positive between 1999 and 2001 and 63% were HPV-positive between 2003 and 2005 [31]. HPV-positive oropharyngeal cancer is considered to be a separate disease with a causal relationship to HPV infection and a good prognosis. Several studies have shown that patients with HPV-positive oropharyngeal cancer, identified through PCR, in situ hybridization or *P16* immunohistochemistry on tumour tissues, have a significantly improved overall and disease-free survival compared with patients with HPV-negative oropharyngeal cancer patients [32,33,34,35,36,37,38,39,40]. In a prospective study involving 253 newly diagnosed or recurrent HNSCC patients, HPV was detected in 25% of patients. A low tumor grade and the oropharyngeal site increased the likelihood of the presence of HPV, respectively [5]. Oropharyngeal tumors are more likely to be positive for HPV (57%) compared with sites other than the tumor site and the oropharynx (14%) and the oral cavity (12%). HPV-positive oropharyngeal carcinoma occurs primarily in the tonsillar of the palatine or tonsils of the tongue. In the tonsils or tongue base, 62% of the tumors were HPV-positive, whereas in other parts of the oropharynx 25% were HPV-positive.

## 3. Pathological Molecular Mechanism in Carcinogenesis

**Figure 1:**
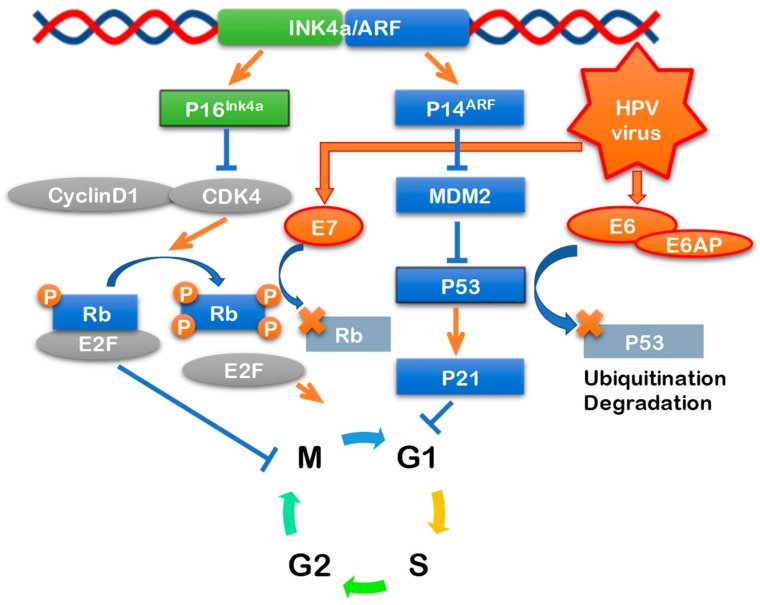
Signaling pathways of high-risk HPV oncogenes. High-risk HPVs encodes two known viral oncogenes. E6 protein inactivates tumor suppressor p53 mediated DNA damage and apoptosis pathway. E7 protein inactivates tumor suppressor pRb mediated cell cycle regulation pathway.

HPV-unrelated HNSCC cigarette smoking and alcohol has p53 mutations [41]. Deletion of 9p21–22 is also observed early in the oncogenic process, and as a result, the function of the tumor suppressor gene *P16* is lost [42]. *P16*INK4a produced by the *P16* gene forms a complex with cyclin 1-cyclin-dependent kinase 4/cyclin-dependent kinase 6 (CDK4/CDK6), inhibits phosphorylation of Rb, and inhibits transcription factor E2F-related cell rotation (pRB pathway) [43]. In HPV-associated head and neck cancer, wild-type p53 is present and mutations occur at a rate of only 10% or less. However, HPVE6 inactivates p53 resulting in a decrease in function. Furthermore, there is no deletion of *P16* in these tumors. Since HPVE7 inactivates phosphorylated Rb, which controls cell cycling of host cells, control of E2F is inhibited [44] and *P16* is overexpressed. *P16* is a tumor suppressor gene encoding a CDK repressor, which inhibits the complex formation of cyclin D1 and cyclin-dependent kinase (CDK) 4/6. Cyclin D1 and CDK 4/6 complex promotes cell cycling through the release of E2F via phosphorylation of the Rb protein, whereas the Rb protein/E2F complex also suppress the transcription of *P16*, so that when HPV-E7 inactivates the Rb protein, *P16* is overexpressed (Figure 1) [45].

Thus, the phenotype at the molecular level is completely different between HPV-positive and HPV-negative cancer of the head and neck. Various methods have therefore been employed for the detection of HPV in head and neck cancer such as consensus primer or type-specific PCR, real-time PCR, in situ hybridization, and serum antibody assays. For cervical cancer screening, accepted international guidelines recommend using hybrid capture II (QIAGEN) and PCR (GP 5/GP 6). 

**Figure 2:**
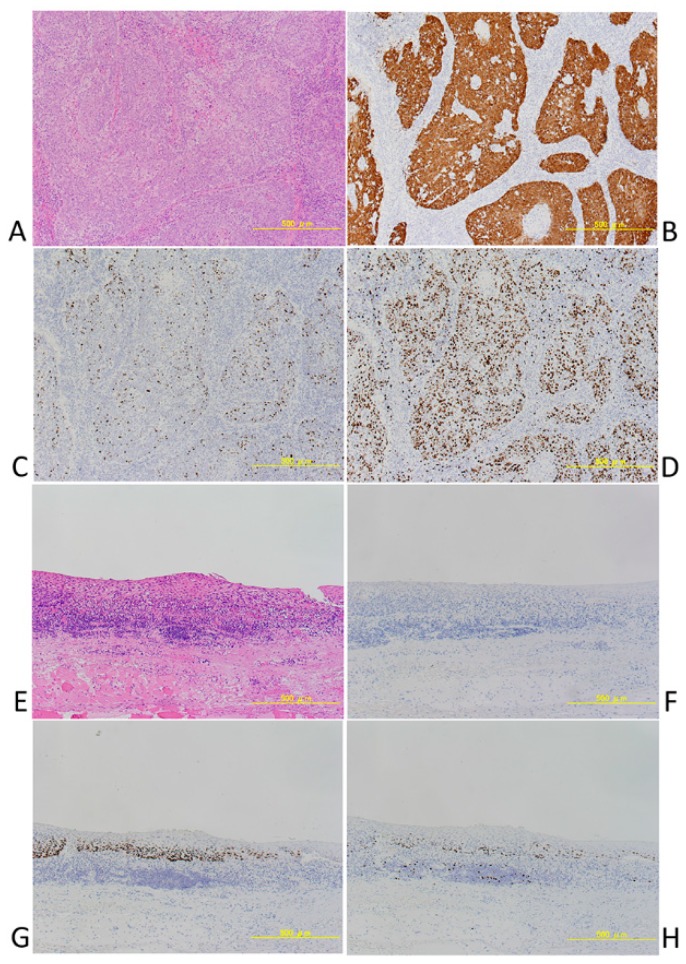
(**A**–**D**) A case of *P16* positive squamous cell carcinoma of Oropharynx. (**A**) HE, (**B**) *P16*, (**C**) p53, (**D**) MIB-1. (**E**–**H**) A case of *P16* positive squamous cell carcinoma of Oropharynx. (**E**) HE, (**F**) *P16*, (**G**) p53, (**H**) MIB-1.

*P16* immunohistochemistry is also useful as a surrogate maker for HPV infection detection, especially in head and neck cancers. *P16* immunohistochemistry has 100% sensitivity and 79% specificity; it is the gold standard for HPV detection, based on HPV 16 E6 and E7 mRNA, in head and neck cancer specimens and is useful as a surrogate maker for clinical HPV detection. *P16* is also a useful molecular marker for judging prognosis and is a component of the WHO classification scheme, described below. *P16*-positive and *P16*-negative HNSCCs could clearly be distinguished in our specimens (Figure 2).

A general consensus has been achieved for the definition of HPV associated tumors that require expression of the virus oncogenic proteins E6 and E7 that are involved in neoplastic transformation of infected cells. However, the integration of HPV-DNA into the host cell genome confirms the belief that it is an essential step for viral oncogene expression in oropharyngeal cancer, as in the case of cervical cancer. Regardless of the process leading to oncogene expression, HPV E6/E7 mRNA identification based on DNA or protein expression, for patient stratification and epidemiologic purposes, is considered a gold standard for HPV-related classification [46,47]. More accessible strategies are generally accepted. In the examination of the pathological specimen, the detection of HPV-DNA using PCR and ISH examination are typically used together with immunohistochemistry of *P16*. Various new methods have been examined for HPV testing in head and neck cancers [48]. When infected cells become malignant, HPV DNA remains in the nucleus, and viral oncoproteins, in particular against E6, are detected in virtually all cases of HPV-driven OPC cases [49,50]. There are reports that HPV-DNA and HPV16 E6 antibodies in oral and in body fluids can be used for detection of HPV-infected head and neck cancers and prediction of the risk of recurrence [51]. 

Studies using next-generation sequencing of HNSCCs have also been reported in recent years. Matthias Lechner did 20 HPV+ and 20 HPV-laser capture microdissection pharyngeal carcinoma studies. HPV-positive and HPV-negative oropharyngeal cancers are divided into two different subgroups. A TP53 mutation was detected in 100% of HPV-negative cases and invalidation of G1/S checkpoint by CDKN2A/B deficiency and/or CCND1 amplification was shown to occur in the majority of HPV tumors [52]. In a study that examined the somatic mutations of 279 HNSCCs performed in 2015, In HPV-positive oropharyngeal carcinoma, deletion of TRAF3, activation mutation of PIK3CA, and amplification of E2F1 were observed. In HPV-negative HNSCC, subsets recognizing simultaneous mutations of CASP8 with amplicons on 11q with CCND1, FADD, BIRC2, and YAP1, or with HRAS, were observed. Either type of tumor was abnormal with respect to target cell cycle, death, NF-κB and other oncogenic pathways [53]. In a study of 92 cases of HNSCC using the next generation sequencer, TPK 53was the most common mutation, occurring in 47 (51%) patients followed by CDKN 2A (*n* = 23, 25%), CCND 1 (*n* = 22, 24%), and PIK 3 CA (*n* = 19, 21%). Changes in TPV, CDKN2A, and CCND1 genes occurred more frequently in HPV-negative tumors, but the total amount of mutations was similar between HPV-negative and HPV-positive tumors. HPV-positive tumors were significantly associated with immune-related genes compared to HPV-negative tumors. Mutations in NOTCH1 (*p* = 0.027), CDKN2A (*p* < 0.001), and TP53 (*p* = 0.038) were significantly associated with decreased overall survival. FAT1 mutation was highly enriched in cisplatin responders and targetable alterations such as PIK3CA E545K and CDKN2A R58X were found in 14 patients (15%) [54]. 

As an emerging technology, liquid biopsies, involving the use of a small amount of DNA and mRNA collected from blood samples, have been used in recent cancer studies. Allen et al. [55] reported a novel in vitro diagnostic approach in which miRNA is examined from exposed cancer cells using sera from HNSCC patients. Of 377 miRNAs detected, 16 different miRNAs were found to be differentially expressed when comparing cells exposed to serum from HNSCC versus healthy individuals. Real-time PCR analysis revealed that serum from HNSCC patients downregulated the expression of 5 genes involved in carcinogenesis and 2 of these—P53 and SLC2A1—are direct targets for the detected miRNAs. This technique has potential for a new therapeutic approach using tumor-specific cell lines or single cell in vitro assays, and the possibility of more specific diagnosis could facilitate individual treatment and early detection of primary tumors or metastasis.

## 4. Classification and Prognosis of Head and Neck Cancers

In general, HPV-positive oropharyngeal carcinoma is highly susceptible to radiation and anticancer drugs and has a better prognosis compared with HPV-negative cancer. HPV-negative oropharyngeal carcinoma is caused by the disappearance of function due to p53 gene mutation [56,57,58,59]. In a prospective phase II clinical trial of pharyngeal and laryngeal cancer patients by the Eastern Cooperative Oncology Group, 63% of 60 cases were HPV 16 positive, and all of the HPV16-positive cases were also positive for *P16*. For HPV-positive and HPV-negative cancers the respective response rates were 84% versus 57%, the 2-year progression-free survival rates were 86% vs. 53%, the overall 2-year survival rates were 95% vs. 62%, and the prognosis for HPV-positive cancer was significantly better [37]. Previous reports have examined the prognosis of HPV in relation to *P16*, and the outcomes of oropharyngeal cancer in relation to tobacco exposure. In a phase III study involving 400 oropharyngeal carcinomas, the 2-year progression-free survival rates for HPV-positive and HPV-negative cancer were 72% vs. 51% and the 2-year overall survival rates were 88% vs. 67%. Due to differences in prognosis, squamous cell carcinoma of the head and neck was classified in the new WHO scheme as HPV-negative or HPV-positive. Classification by *P16* immunostaining or HPV testing is recommended. In addition, some recently proposed classification schemes are based on the EGFR status according to 2 categories: HPV-positive/*P16* positive squamous cell carcinoma and HPV-negative/*P16* negative squamous cell carcinoma [60]. Cetuximab, a monoclonal antibody that inhibits the function of EGFR, is known to have efficacy in colorectal cancer [61,62] and head and neck cancer [63,64], and it was also shown to be more specific and cost-effective for these types of cancers.

## 5. Prospects for Treatment 

For the reasons outlined herein, HPV virus is expected to be a therapeutic target in the treatment of human cancer [65] We believe that the prognosis for cervical cancer can be greatly improved through the implementation of methods for early detection such as cytodiagnosis, HPV screening, and *P16* immunostaining, Furthermore, HPV vaccination may also be useful for preventing HPV-related cancers other than cervical cancer. For example, most cases of oropharyngeal carcinoma are caused by HPV 16 (about 90%) and HPV 18, and HPV vaccination for this condition can be expected to have a greater disease-suppressing effect than in cervical cancer. Randomized trials have provided strong evidence for high efficacy of the 2 FDA-approved VLP vaccines: the bivalent HPV16/18 vaccine (CervarixÒ, GlaxoSmithKline Biologicals, GlaxoSmithKline plc, Brentford, UK) and the quadrivalent HPV 6/11/16/18 vaccine (Gardasil™, Merck Sharp and Dohme, Merck & Co., Kenilworth, NJ, USA) against cervical, vaginal, and vulvar HPV16/18 infections and related diseases, and against anal HPV16/18 infections in women [66,67,68,69,70]. One of the methods proposed for prevention of HPV-related oropharyngeal cancer is vaccination. The US Centers for Disease Control Advisory Committee on Immunization Practices (ACIP) has recommended HPV vaccination for females and males between the ages of 11 and 12 years, starting as early as 9 years, with booster doses at up to 26 and 21 years for females and males, respectively. In order for the vaccination to be effective in preventing oropharyngeal cancer, the protective effect must last for at least 2 decades, and ongoing studies have shown no waning of systemic antibodies at 8 years after vaccination. However, in June 2013, the Ministry of Health, Labour and Welfare of Japan suspended proactive recommendation of the vaccine after unconfirmed reports of adverse events. To investigate any potential association between the vaccine and reported symptoms, the Nagoya City Council conducted a questionnaire-based survey. The anonymous postal questionnaire investigated the onset of 24 symptoms, associated hospital visits, frequency, and influence on school attendance. A total of 29,846 residents responded. No significant increase in occurrence of any of the 24 reported post-HPV vaccination symptoms was found. The results suggest no causal association between the HPV vaccines and reported symptoms [71]. 

Also, Reduction surgery or minimally invasive treatment should be considered in cases of HPV virus-related oropharyngeal carcinoma. Although limited to the T1 and T2 stages of oropharynx cancer, transoral robotic surgery approved by the FDA since 2009. In addition, many reduction surgeries and post-operative adjuvant therapies based on pathologic staging are being studied [72,73,74]. 

As mentioned above, it is known that EGFR is expressed in HNSCCs, and combination use of cetuximab and radiation is, therefore, being studied for treatment instead of standard cisplatin therapy [38,61,64,75,76,77,78]. Several researchers have hypothesized that radiation dose reduction is feasible and safe for some HPV-positive patients when induction chemotherapy (IC) is used for patient selection. Three points have been raised to support this assertion. First, HPV-positive HNSCCs are considered to be more radiosensitive than HPV-negative HNSCCs [79]. Second, doses comparable to the supplemental radiation dosage is sufficient for the treatment of patients with asymptomatic disease [80]. Finally, the response to chemotherapy can predict the future response to subsequent radiation therapy. Chen et al. [79] conducted an arm Phase II trial (NCT 01716195) in 44 patients with stage III/IV *P16*-positive OPCs treated 2 cycles of IC (paclitaxel and carboplatin was for 21 days), followed by radiation and paclitaxel. The radiation dose was also reduced in complete or partial responders (54 Gy, *n* = 24), and even in patients with partial or no response (*n* = 20, 60 Gy instead of standard 70 Gy). Except for 1patient, all patients completed the IC. Two years PFS and local region control were 92% (95% CI 77–97) and 95% (95% CI 80–99), respectively. The two-year degree of freedom for grade 3 or adverse events of worsening mucosa and esophagus was 85% (95% CI 80–90) in patients treated with 54 Gy. These results were achieved with doses reduced by 15–20% compared with these in the ECOG 2399 trial using the same protocol, except that a dose of 70 Gy, dose was used as a standard chemoradiotherapy regimen. Besides that, Removal of chemotherapy and Alternative to the “conventional” photon beam therapy are considered as new treatment methods [80].

Differences in the prognosis and etiologic mechanisms of HPV-related head and neck cancer from conventional head and neck cancers (mostly HPV-negative) suggest that the detection of HPV may significantly change the future diagnosis, treatment, and management [31]. HPV not only plays a role in the development of pharyngeal cancer but is also involved in 23.5% of oral cancer and 24% of laryngeal cancer cases, suggesting that indications for HPV vaccination could be expanded to also include oral and laryngeal cancer [37].
